# Human inner ear blood supply revisited: the Uppsala collection of temporal bone—an international resource of education and collaboration

**DOI:** 10.1080/03009734.2018.1492654

**Published:** 2018-09-11

**Authors:** Xueshuang Mei, Francesca Atturo, Karin Wadin, Sune Larsson, Sumit Agrawal, Hanif M. Ladak, Hao Li, Helge Rask-Andersen

**Affiliations:** a Department of Surgical Sciences, Section of Otolaryngology, Uppsala University Hospital, Uppsala, Sweden;; b Department of Otolaryngology, Peking University Shenzhen Hospital, P.R. China;; c Department of Diagnostic Radiology, Uppsala University Hospital, Uppsala, Sweden;; d Department of Surgical Sciences, Section of Orthopedics, Uppsala University Hospital, Sweden;; e Department of Otolaryngology-Head and Neck Surgery, Western University, Canada;; f Department of Medical Biophysics and Department of Electrical and Computer Engineering, Western University, Canada

**Keywords:** Human, micro-computerized tomography, synchrotron phase contrast imaging, temporal bone, Uppsala collection

## Abstract

**Background:** The Uppsala collection of human temporal bones and molds is a unique resource for education and international research collaboration. Micro-computerized tomography (micro-CT) and synchrotron imaging are used to investigate the complex anatomy of the inner ear. Impaired microcirculation is etiologically linked to various inner ear disorders, and recent developments in inner ear surgery promote examination of the vascular system. Here, for the first time, we present three-dimensional (3D) data from investigations of the major vascular pathways and corresponding bone channels.

**Methods:** We used the archival Uppsala collection of temporal bones and molds consisting of 324 inner ear casts and 113 macerated temporal bones. Micro-CT was used to investigate vascular bone channels, and 26 fresh human temporal bones underwent synchrotron radiation phase contrast imaging (SR-PCI). Data were processed by volume-rendering software to create 3D reconstructions allowing orthogonal sectioning, cropping, and soft tissue analyses.

**Results:** Micro-CT with 3D rendering was superior in reproducing the anatomy of the vascular bone channels, while SR-PCI replicated soft tissues. Arterial bone channels were traced from scala vestibuli (SV) arterioles to the fundus, cochlea, and vestibular apparatus. Drainage routes along the aqueducts were examined.

**Conclusion:** Human inner ear vessels are difficult to study due to the adjoining hard bone. Micro-CT and SR-PCI with 3D reconstructions revealed large portions of the micro-vascular system in un-decalcified specimens. The results increase our understanding of the organization of the vascular system in humans and how altered microcirculation may relate to inner ear disorders. The findings may also have surgical implications.

## Introduction

Jan Stahle and Herrmann Wilbrand (professors in Otorhinolaryngology and Oto-radiology at Uppsala University) introduced the idea of developing a collection of human inner ear molds. The purpose of this collection was to describe the minuscule structures of the human inner ear, such as the vestibular and cochlear aqueducts, and to observe their roles in Meniere’s disease (1–3). These small channels are also associated with the accessory canals housing blood vessels (4–6), which are believed to play a role in the circulation of the inner ear fluids (2). Wilbrand and collaborators had a deep interest in temporal bone anatomy and its radiological appearance. They used polytomography before the era of modern computed tomography (CT). Radio-anatomic correlations were made and a new technique developed to cast human inner ears using methacrylate and silicon with a low shrinkage factor. A large collection was built with 325 corrosion casts at the Department of Radiology (3). Moreover, 113 temporal bones were collected, of which 85 were micro-dissected focusing on the inner ear aqueducts (2,7). The casts belong to the Museum of Medical History at the Uppsala University (http://www.medicinhistoriskamuseet.uu.se) but are currently under the care of the Department of Otorhinolaryngology at the Uppsala University Hospital for utilization in national and international anatomy courses and research projects. Due to the progress of cochlear implantation (CI) and other implantable systems, the specimens are used by surgeons to comprehend the complex inner ear anatomy. CI surgery is now performed, even in patients with residual hearing, and understanding of structural variations is essential for inner ear tissue preservation and optimal surgical outcome.

In 2017, several specimens underwent micro-computerized tomography (micro-CT) at Professor Sune Larsson’s Laboratory at the Department of Orthopedics of the Uppsala University Hospital in cooperation with Canadian researchers in London, Ontario, who cooperate with the Bio-Medical Imaging and Therapy (BMIT) facility at the Canadian Light Source in Saskatchewan, which is funded by various Canadian organizations. This facility provides world-class technology with unique synchrotron-specific imaging and therapy capabilities.

Inner ear surgery requires an awareness of the main vascular structures. However, preparation of the human cochlea is challenging due to its complex anatomy and vulnerability and because it is surrounded by the hardest bone in the body, a challenge well-recognized by early anatomists. Modern imaging techniques offer unique possibilities to visualize and reconstruct three-dimensionally (3D) the bone and soft tissues without decalcification, and further revelations concerning this technology have recently been presented (8,9). Here, we describe the use of these techniques in the production of direct virtual 3D inner ear replicas as an alternative to four months of laborious molding. The datasets allow multi-slicing and 3D cropping of microstructures, such as the spirally arranged blood vessels and their origins. These findings may bridge limitations in our understanding of the vascular anatomy of the human labyrinth, due to the specific focus of the present study.

### Human inner ear vascular supply

The human inner ear depends on a vascular supply to maintain fluid homeostasis, ion balance, and metabolic supply. Disruption of the cochlear blood flow leads to instantaneous pathological changes in the inner ear (10,11), and a disturbed microcirculation may be etiologically linked to various inner ear disorders, such as sudden deafness, autoimmunity, presbycusis, noise-induced hearing loss, vestibular neuritis, and Meniere’s disease (12–27).

Like the eye, the inner ear is supplied with an end artery. Its subdivisions trace a path across the perforated bony wall in the fundus to reach the membranous labyrinth with capillary regions. Many earlier vascular supply studies were conducted in animals and humans, revealing large species and individual differences (28–39). Investigators used impressive injection techniques combined with decalcification, sectioning, clearance, and surface preparations. Unfortunately, there was no general consensus among authors on the nomenclature of the inner ear vessels, and the results depended on their ability to completely fill the vascular ramifications.

**Figure 1.**
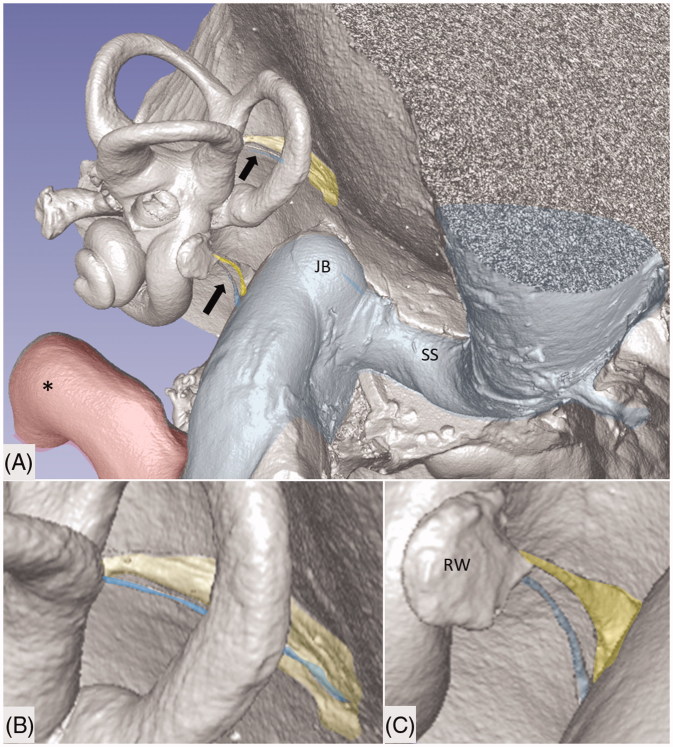
Micro-CT and volume rendering of a plastic corrosion cast of a left human temporal bone. The VA and CA (yellow) with their accessory canals (blue) are seen (arrows). They drain blood from the inner ear and are shown in higher magnification in B and C. *carotid artery; JB: jugular bulb; RW: round window; SS: sigmoid sinus.

In the present study, we used non-invasive, X-ray imaging techniques to trace and visualize the major intra-cochlear blood vessels running in bone channels, including draining outlets. High-resolution synchrotron radiation phase contrast imaging (SR-PCI) with 3D renderings of non-decalcified, post-mortem human temporal bones and micro-CT (μCT, 9 micron pixel resolution) of macerated temporal bones and inner ear molds were used. Datasets were fed into an open software platform for medical image informatics and 3D visualization, including virtual sectioning. This allowed the tracing of separate vascular bone channels and their contents. Arteries could be separated from veins by their convoluted appearance and by following them back from identified vessels, such as the radiating arterioles of the scala vestibuli (SV). Inner ear veins drain mainly through separate accessory channels along the cochlear and vestibular aqueducts (Figure 1).

### History

**Figure 2.**
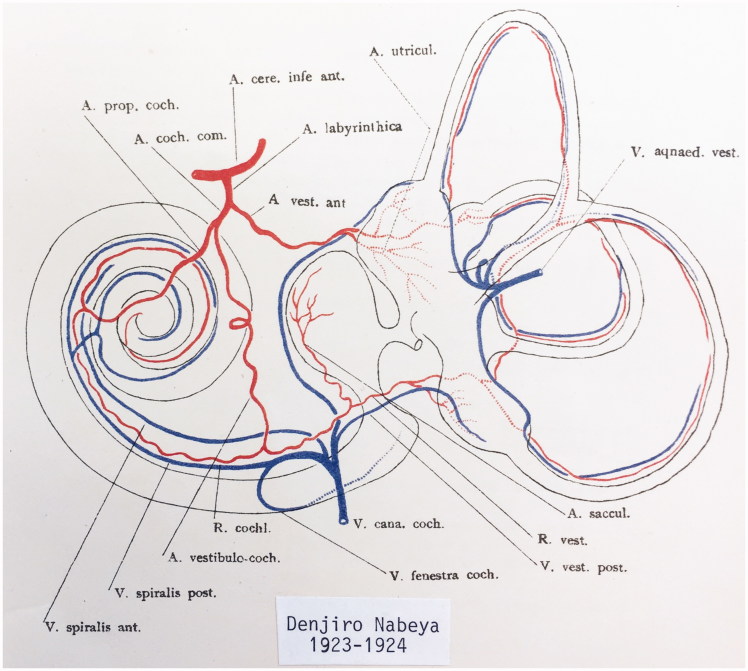
Principal vascular anatomy of the human inner ear by Nabeya (42).

Schwalbe (40) was the first to systematically describe the circulation of the human cochlea, followed by Eichler (41), Siebenmann (5), and Nabeya (42), who used intravascular dye injections. Nabeya focused on the larger vessels in 16 fetuses and 8 adult temporal bones. He found that the inner ear is supplied by only one end artery which he named the labyrinthine artery (LA). The LA mostly derives from the anterior inferior cerebellar artery and most often has three branches within the internal acoustic canal (IAC) (Figure 2), namely, (1) the anterior vestibular artery (AVA); (2) the vestibulo-cochlear artery (VCA); and (3) the cochlear artery (CA). However, authors may use different names for these vessels. The VCA divides into cochlear and vestibular branches which run in opposite directions. The vestibular branch supplies the vestibule and semicircular canals, while the cochlear branch runs spirally along the SV to anastomose with the CA. The cochlear branch mostly supplies the second quarter of the basal turn, but it sometimes supplies the entire cochlea. The CA is said to be the dominating vessel for the cochlea and can be replaced by the cochlear branch of the VCA (41,42). Siebenmann (5) defines this as the ‘tractus spiralis arteriosus’, and Levin (34) describes glomus-like bodies. Various descriptions of the venous drainage exist, but there is no general consensus about the descriptions. The venous routes go along the cochlear and vestibular aqueducts with the inferior cochlear vein and vein of the vestibular aqueduct (VA), respectively. Some authors have described a vein in the IAC, but others have denied it.

Branches of the LA and denominations used here are as follows:

1. Cochleo-vestibular artery (CVA)
  - Vestibular branch
  - Cochlear branch
2. Anterior vestibular artery (AVA)
3. Cochlear artery (CA or spiral modiolar artery)

## Material and methods

### Temporal bone collection

A total of 113 archival unselected human temporal bones from autopsies were analyzed. The specimens were generously provided by the Museum of Medical History at the Uppsala University. The results obtained from this collection were previously published (1,2). Some 35 bones were macerated and un-dissected. The collection also contained 324 plastic and silicone molds made from the labyrinth according to molding techniques described by Wilbrand et al. in 1974 (1).

### Micro-CT

A total of 100 plastic corrosion casts from human temporal bones underwent micro-CT and 3D reconstruction (Figure 1). The bones were scanned with micro-CT (SkyScan 1176; Bruker, Belgium) using the following parameters: 65 kV source voltage, 385 µA current, 9 µm pixel size, 1 mm Al filter, 1 s exposure time, 2 frame averaging, and a 0.30° rotation step. The projection images were acquired over an angular range of 360°, with an angular step of 0.3°. In the resultant images, the image size was 4,000 × 2,672 pixels, and the pixel size was 9 μm. Projections were reconstructed using NRECON ver. 1.7.0.4 (Bruker) software based on the Feldkamp algorithm. A volume-rendering technique was used to present the two-dimensional (2D) projection of a 3D discretely sampled dataset produced by the micro-CT scanner and visualized using the CTvox 3.0 application (Bruker). Opacity and gray scale values were adjusted to create a realistic 3D view which was as similar to the actual bones as possible. Geometric measurements were performed, and images were obtained utilizing a 3D slicer program (Slicer 4.6; www.slicer.org). This 3D slicer program is an open software platform for medical image informatics, image processing, and 3D visualization (43). The visualization of the surface anatomy of the temporal bone was performed with micro-CT. The images were resized at a 4:1 ratio before 3D reconstruction because of hardware and software limitations. Opacity and gray scale values were adjusted during the volume rendering. The application displays reconstructed slices as 3D objects and provides a realistic 3D visualization of scanned objects. Virtual sectioning of the petrous bone revealed internal areas of the bone. The 3D modeling software was equipped with tools which allow geometric measurements in 3D. Different anatomic variations can therefore be described. The technique also allows orthogonal sectioning or cropping techniques.

### SR-PCI

The SR-PCI technique used was recently described by Elfarnawany et al. (8) and Koch et al. (44). A total of 16 fresh-frozen and then fixed adult cadaveric temporal bones were used in this study. All specimens were obtained with permission from the body bequeathal program at Western University, London, Ontario, Canada, in accordance with the Anatomy Act of Ontario and Western University’s Committee for Cadaveric Use in Research. After thawing, a cylindrical cutter was used to core a sample (40 mm diameter, 60 mm length) from the middle ear of each temporal bone. The samples were fixed in a 4F1G (3.7% formaldehyde and 1% glutaraldehyde in phosphate buffer) bath for 5 d. The samples were rinsed twice and dehydrated using an ethanol series (50%, 60%, 70%, 80%, 90%, 95%, and 100%). No additional processing (i.e. staining, sectioning, or decalcification) was performed on the samples. Sample fixation eliminated the risk of degradation over the two-month time difference between imaging sessions and scanning. Samples were transferred to the imaging facilities in motion-proof containers to prevent damage during shipping.

The PCI technique was in-line PCI, a setup similar to conventional radiography. It consists of an X-ray source, a sample, and a detector with no other optical elements. The detector is placed at a distance from the sample which allows the phase-shifted beam to interfere with the original beam and produce measurable fringes. The fringes correspond to surfaces and structural boundaries of the sample (edge enhancement) compared with a conventional radiogram. To obtain SR-PCI images, each sample was scanned using the BMIT 05ID-2 beamline at Canadian Light Source Inc. in Saskatoon, SK, Canada. This device provides an SR beam produced by a superconducting wiggler source (45). The beam is filtered using a monochromator and yields an energy bandwidth of ΔE/E = 10^−3^ over an energy range of 20–150 keV (8). The imaging setup, installed at the beamline length of 55 m from the source, consists of a sample stage and a charge-coupled device-based detector system, which are both placed on a vibration isolation table. The distance between the sample and detector was 2 m, and the photon energy was 47 keV. Motorized alignment stages were used to align the sample and detector for high-resolution tomography. The detector was an AA-60 beam monitor coupled with a C9300-124 camera (Hamamatsu Photonics, Shizuoka, Japan), which has a 12-bit resolution and an effective pixel size of 9 × 9 µm^2^. The imaging field of view was set to 4,000 × 950 pixels corresponding to 36.0 × 8.6 mm; 3,000 projections over 180 rotations were acquired per view. The 3D image volume had an isotropic voxel size of 9 µm. The acquisition time to capture all projections per view was ∼30 min. While CT imaging is absorption contrast-based, PCI can potentially be combined with synchrotron imaging to improve soft-tissue contrast while maintaining accurate visualization of bone. Conventional absorption contrast-based CT depends on the attenuation of X-rays, whereas, in PCI, the phase shift caused by the sample is transformed into detectable variations in X-ray intensity. PCI can provide edge enhancement by emphasizing the contrast between boundaries of different structures in the image. The results demonstrate that SR-PCI can be used to visualize both bone and soft tissue simultaneously.

## Results

### General anatomy

**Figure 3.**
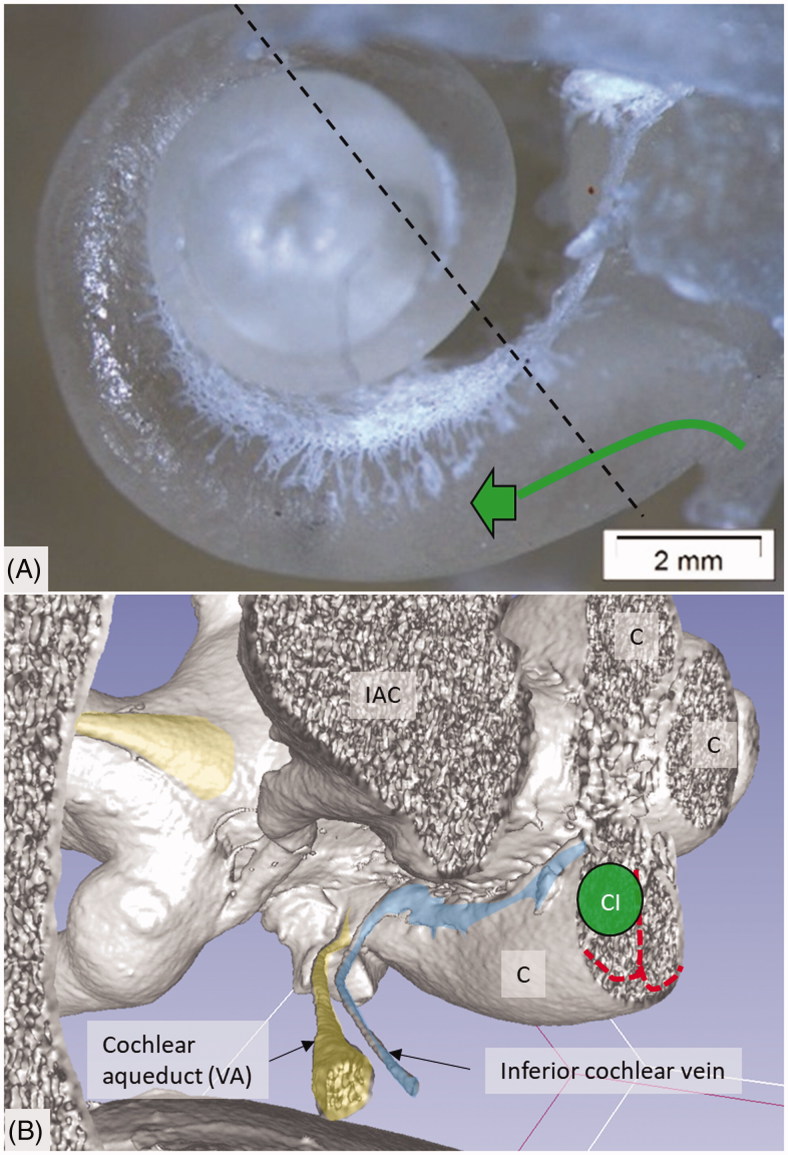
(A) Plastic corrosion cast of a left human cochlea. Radiating arterioles of the basal turn of the cochlea can be observed. The route for electrode insertion at the CI is shown in green at arrow. (B) Micro-CT and 3D rendering of a plastic mold from a left human temporal bone (medial view). The level of cropping is shown (interrupted line). The inferior cochlear vein channel is seen (blue), as well as the cochlear aqueduct. The vein drains blood from the cochlea and runs parallel to the cochlear aqueduct. C: cochlea; CI: cochlear implantation; IAC: internal acoustic canal.

**Figure 4.**
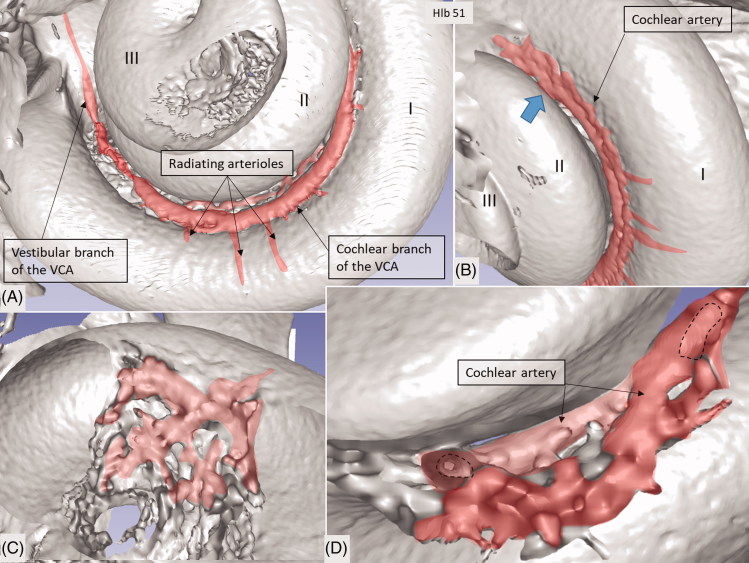
Micro-CT and 3D rendering with surface enhancement of the inner labyrinth in a right macerated human temporal bone. (A) The tractus spiralis arteriosus (red) supplying the SV with radiating arterioles is seen (Roman numerals represent cochlear turns). The VCA branches into the vestibular and cochlear branches which run in opposite directions. (B) The cochlear branch runs spirally and anastomoses with the CA (blue arrow) at the upper segment of the first cochlear turn. The size and anatomy vary greatly among different temporal bones. (C and D) Anastomosis shown between the cochlear branch of the CVA and the CA. Several tributaries project into the modiolus, supplying the spiral ganglion and spiral lamina.

Micro-CT scans of inner ear molds with 3D reconstruction displayed the arterial bone channels and the drainage veins along the aqueducts (Figure 1). Furthermore, the radiating arterioles of the SV could be followed to the arteries between the first and second turns. By cropping, the modiolar area could be disclosed and analyzed in more detail (Figures 3 and 4). Micro-CT of macerated temporal bones with a surface enhancement algorithm and removal of endosteal bone made it possible to analyze these vessels in even greater detail along the cochlear spiral. The arteries could be identified by following the radiating arterioles back to the main trunks. They also had a coiling structure typical of arteries. The rich vascular space at the basal turn also contained veins and could be characterized as an *area vasculosum.*

### Tractus spiralis arteriosus

**Figure 5.**
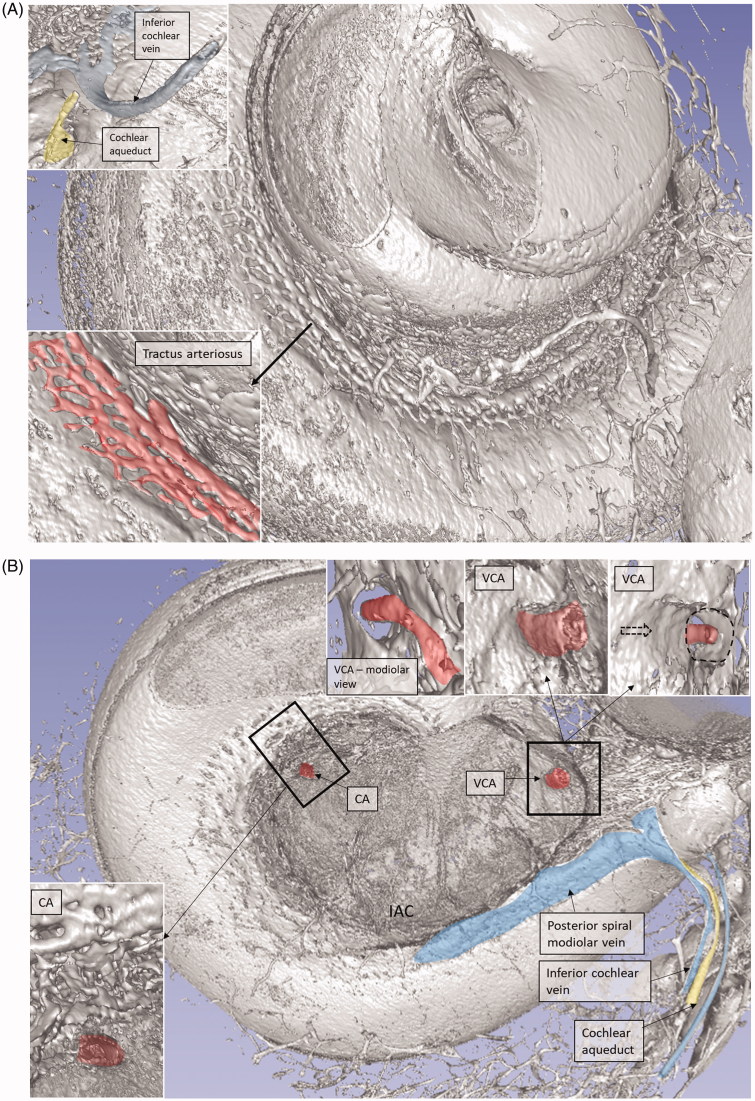
SR-PCI image of a left human cochlea. (A) The tractus spiralis arteriosus is seen between the first and second turn (lower inset). The draining inferior cochlear vein is identified with the merging modiolar spiral veins. (B) The soft tissues in the IAC (yellow) and the inferior cochlear vein are reproduced (blue). The entrances of the VCA and CA can be traced. Note that the VCA crosses through a structurally modified channel.

**Figure 6.**
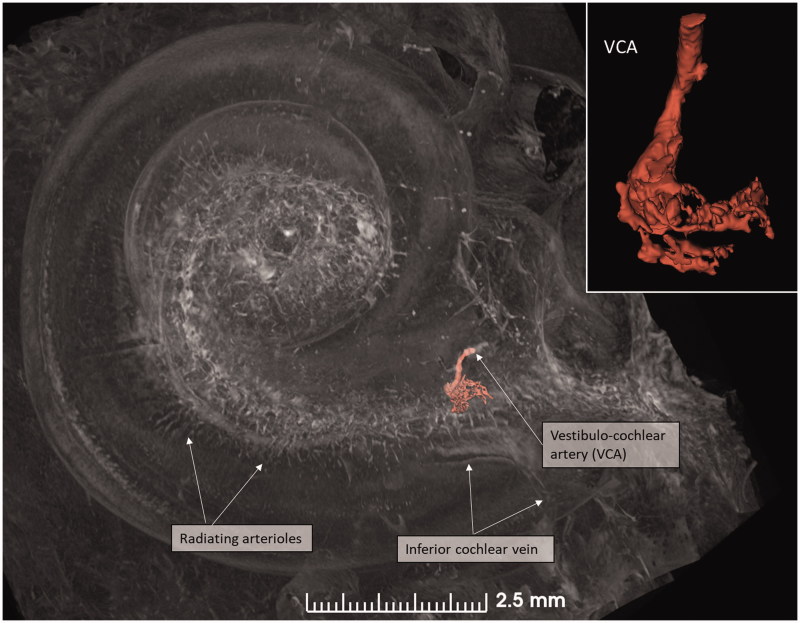
SR-PCI image of a right human cochlea. The VCA was segmented and is shown in higher magnification in the inset. After entry into the modiolus, it divides into several small arteries or plexus.

**Figure 7.**
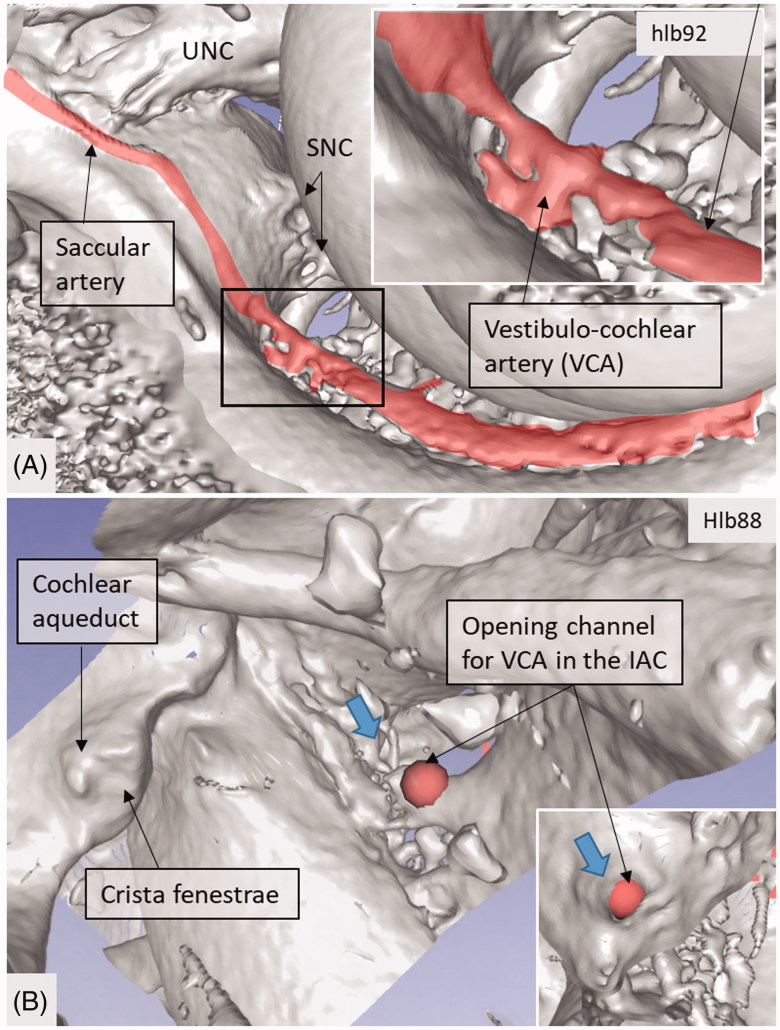
(A) Micro-CT of a right cochlea showing the VCA dividing into vestibular and cochlear branches running in opposite directions. The framed area is magnified in the inset. (B) The VCA is traced in the IAC using a red marker. SNC: saccular nerve canal; UNC: utricle nerve canal.

**Figure 8.**
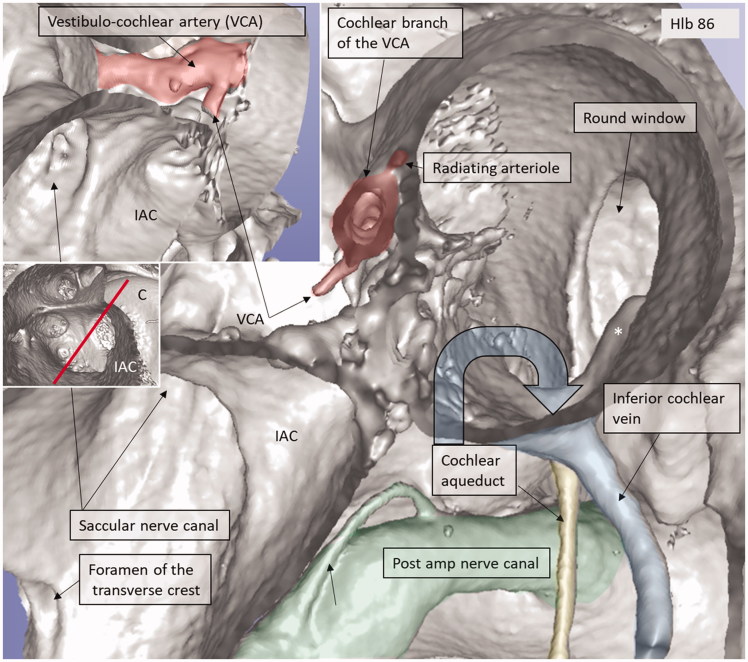
3D view of a cropped human cochlea at the level of IAC. Removal of bone and surface enhancement reveal vascular structures and nerve canals. The basal turn is sectioned horizontally near the RW. The cochlear aqueduct and inferior cochlear vein are seen. A radiating arteriole is identified and can be followed to the main stem of the cochlear branch of the VCA. The VCA is smaller than usual, suggesting that the main arterial supply to the basal turn is through the CA. C: cochlea.

An anastomosing arterial network was identified between the first and second turns (Figure 5(A)). This system was named the tractus spiralis arteriosus foraminulentus earlier by Siebenmann (5) and Nabeya (42). The 3D reconstructions showed that this impressive arterial system consists of a band of branched arteries which sends radiating arterioles to the SV at all turns. Here, its source was the VCA which reached the area through a basal hole in the fundus lamina cribrosa (Figure 5(B), Figures 6, 7, and 8) and a descending spiral branch of the CA. SR imaging showed a soft tissue collar around the artery at the bony opening. The VCA divided into two branches going in opposite directions (Figure 7(A)). A vestibular branch ran along the surface of the saccule (where it divided into a branch to the superior vestibular nerve canals [SVNCs] and one to the saccule) and a cochlear branch, which spiraled along the modiolus and anastomosed with the CA (Figure 4). The CA entered between the first and second turn near the upper basal turn. The cochlear branch varied greatly in size. Sometimes, the cochlear branch was small, and the main supply to the arterial system was from the CA. The principal organization of this arterial supply adhered to the description by Nabeya (42). The arterial system could also be viewed with the SR technique and with the endosteal bone removed. The modiolar space between the first and second turn varied in size depending on the coiling of the first cochlear turn. The tractus arteriosus continued apically along the cochlear spiral. Connections between the CA and the tractus arteriosus bone channels could be traced through a 3D tracking system algorithm on serial X-ray sections. Ramifications were followed into the modiolus and inter-scalar septa, which made it possible to trace the CA channels to the fundus region. Branches sent to Rosenthal’s canal and the spiral lamina could also be identified.

### AVA and foramen of the transverse crest

**Figure 9.**
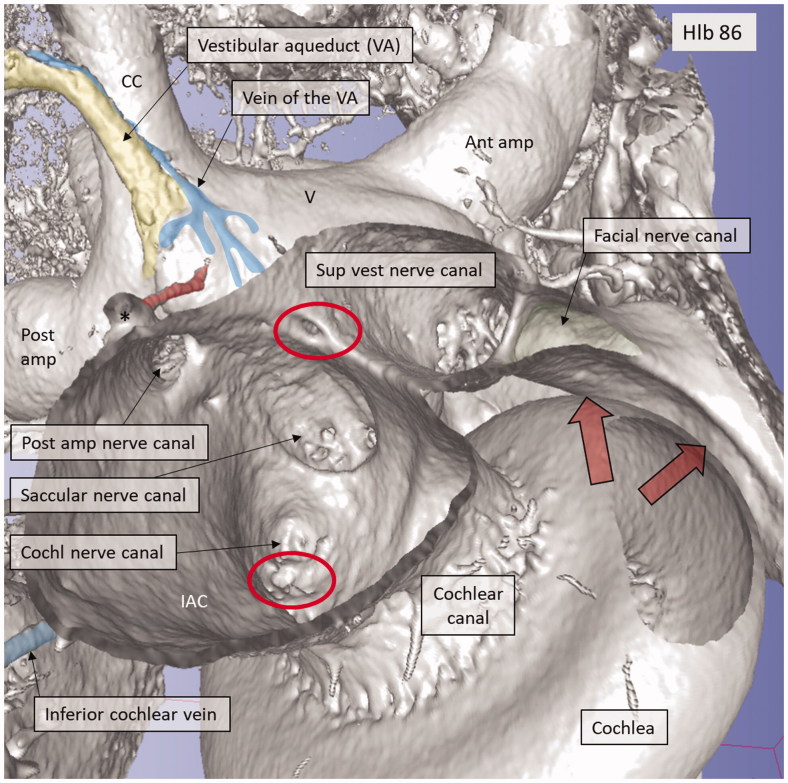
Micro-CT and 3D rendering of a left human temporal bone after virtual molding of the labyrinth using surface enhancement and medial cropping. The technique allows visualization of the labyrinthine cavities. Medial 3D view of a cropped human labyrinth at the level of IAC. The removal of bone reveals openings of the nerve canals in the fundus, as well as the medial surface of the vestibule (V) with common crus (CC), anterior ampulla (Ant amp), and the posterior ampulla (Post amp), together with the vestibular aqueduct (VA) and the vein of the VA. Note the impression of the facial nerve canal caused by the cochlea (red arrows). The inferior cochlear vein can be seen. The foramen of the TC (upper red circle) and the region for the entrance of the VCA (lower red circle) can be seen.

**Figure 10.**
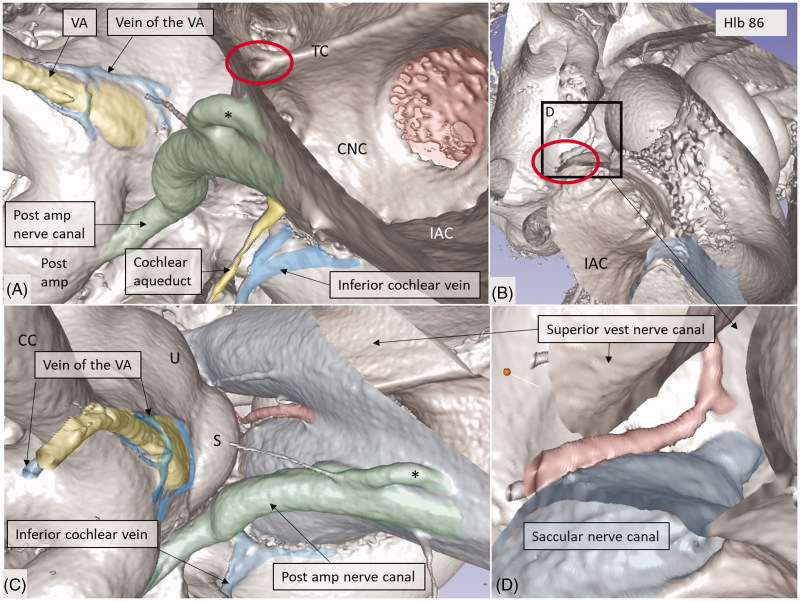
Micro-CT and 3D rendering of a left human temporal bone after virtual molding of the labyrinth using surface enhancement and coronal cropping. (A) The 3D view of a cropped human labyrinth at the level of IAC. The removal of bone and surface enhancement reveal both vascular structures and nerve canals. The cochlear aqueduct and the inferior cochlear vein can be seen. (B) The cochlea is sectioned horizontally near the foramen of the TC (red circle). This channel seems to house a vessel or artery which supplies both the utricle and the saccule. Framed area is shown in higher magnification in (D). (C): Lateral view of the IAC showing the channel of the TC (red). The VA and the accessory canal housing the vein of the VA are identified. CNC: cochlear nerve canal; CC: common crus; S: saccule; TC: transverse crest; U: utricle; *accessory singular nerve canal.

**Figure 11.**
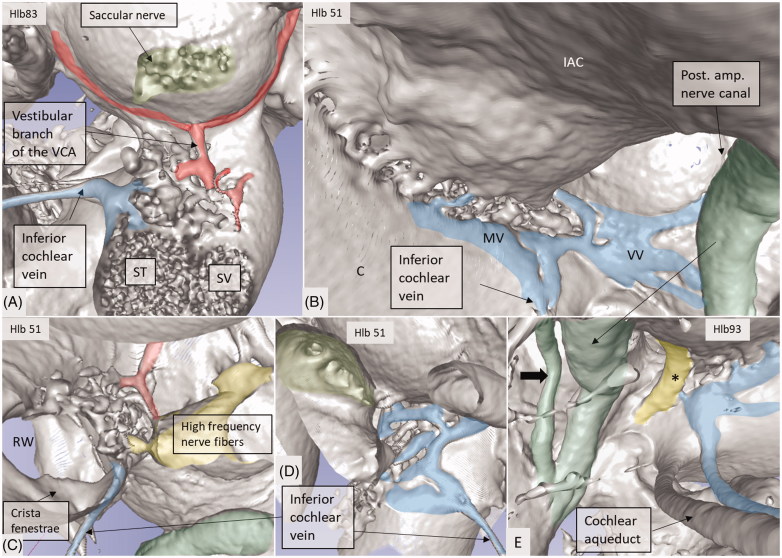
Micro-CT and 3D rendering show anatomic variations of the inferior cochlear vein. (A) Medial view of the basal turn and saccule after cropping. Note the close relationship between the artery and veins. ST: scala tympani; SV: scala vestibuli. (B) Inferior view of the C and IAC. Modiolar veins (MVs) and vestibular veins (VVs) coalesce and drain into the inferior cochlear vein. (C) The inferior cochlear veins run in the floor of the ST near the opening of the cochlear aqueduct and the RW. A channel housing the high-frequency nerve fibers can be seen. (D) Veins draining in the base of the C. Entrance of the saccular nerves are stained green. (E) The relationship between the inferior cochlear vein (blue) and the channel for the high-frequency nerve fibers (*) are seen.

The foramen of the transverse crest (TC) was ubiquitously found and varied in size (Figures 9 and 10). The channel could be followed to the saccule and utricle, and branches continued along the anterior and lateral ampulla nerve canals. The canal was thought to house an artery representing the AVA or a branch of it, which is the first branch of the LA. However, this could not be unequivocally proven. Several bone channel openings were found in the lateral wall (LW) of the IAC, especially around the opening of the singular nerve canal (SiNC). Some of these channels ran to the posterior ampulla close to the ampulla nerve (Figure 10(A, C), Figure 11(E)).

### Venous drainage

The draining veins of the vestibular organ and cochlea were identified (Figure 1, Figure 3(B), Figure 5(A) upper inset, Figure 5(B), Figures 8, 9, and 11). The inferior cochlear vein exits at the floor of the scala tympani (ST) near the opening of the cochlear aqueduct and the round window (RW). Several draining veins merge here, with two major kind of variations, depending on how the anterior and posterior spiral modiolar veins develop and on the posterior and anterior vestibular veins (Figure 11). Occasionally, veins of the RW could be detected. The posterior spiral veins received several tributaries from the lateral scala tympani wall. A draining vein in the IAC could not be evaluated with the current techniques. Cochlear veins converged and concerted in an *area vasculosum* (our term) near the entering arteries at the basal turn of the cochlea.

## Discussion

The Uppsala collection offers unique opportunities for surgeons to study the 3D anatomy of the human temporal bone with variations (7,9,46). Recently, in this journal, we presented the complex anatomy of the *hook* region of the cochlea, together with the RW and basilar membrane, using SR imaging, which is relevant for cochlear surgeons (9). Here, we present, for the first time, the 3D reproduction of the human vascular supply using SR imaging and micro-CT. Knowledge about the intricate vascular system is essential in modern inner ear surgery. Major arteries and veins were identified in un-decalcified temporal bones, while capillary areas were challenging to identify. Volume rendering of bone configurations was superior with micro-CT, while SR imaging replicated soft tissues. Bone transparency disclosed several canaliculi within the otic capsule, which occasionally blurred labyrinth exposure, but this could be resolved by cropping. The specimens are also valuable when applying light and electron microscopy (47,48).

### Tractus spiralis arteriosus

The tractus arteriosus formed an impressive communicating system of branched arteries, reflecting the extensive cochlear blood supply, including the LW which generates the endo-cochlear potential. Basally, the vascular plexus originated from the CVA, which often anastomosed with a CA. This connection may secure an unceasing supply in case obstruction occurs in any of the branches. This has clinical relevance, since the lack of anastomoses could make regions more susceptible to damage. The results substantiate Nabeya’s findings (42) of arterial entry routes. In his extensive work on blood supply, including comparative species analyses, he managed to trace the blood vessels by elaborate vascular injections and sectioning. His work was partly executed in Professor George Schambaugh’s laboratory in Chicago in the USA. Nabeya described two types of arterial supply to the human cochlea. In type I, the CA dominates and supplies the lower basal turn of the cochlea. The cochlear branch of the VCA is small, and the cochlear and vestibular branches derive mainly from the CA. In type II, the VCA dominates and supplies the basal part of the cochlea and anastomoses further up with the CA. Both of these systems were verified in this study. Whereas the AVA and CA follow their respective nerves to the inner ear, the VCA perforates the lamina cribrosa between the saccular nerve and the high-frequency cochlear fibers at the basal end of the cochlea. The vessel runs through a narrow bone canal and can be obstructed under various conditions, such as increased intra-cranial pressure. The stent-like soft tissue embracing the VCA at the fundus orifice could sustain openness and assure unimpeded circulation into the cochlea.

The unique arterial plexus along the modiolus serves to supply the cochlea with a large amount of oxygenated blood. Levin (34) and Malan (49) described vascular convolutions with specialized arterio-venous connections. Schwalbe (40) has already described glomus-like structures and speculated on these functions to even out pulsatile pressure waves. Such pulses could negatively influence the sensitive mechanoreceptors and hearing. Furthermore, Balogh and Koburg (50) described a ‘plexus cochlearis’ in the modiolus with very high metabolic turnover rates, even higher than those of the spiral ganglion. This resembled the choroid plexus with extending spider-web-like connective tissue processes, and they speculated on a secretory function. We found that veins traversed the arterial system in close proximity before joining the inferior cochlear vein (ICV). We speculate that the vascular convolutes could monitor gas exchange and pressure alterations and thereby regulate cochlear blood flow via this well-vascularized zone. Thus, further analyses of the vascular plexus are indicated.

### Vestibular system

The vestibular system receives arterial tributaries from both the AVA and the vestibular branch of the VCA. This suggests that the capillary regions of the sensory epithelia of the saccule, utricle, and anterior and lateral ampulla may have a dual supply. The origin of the AVA was difficult to assess using the present techniques. We recently identified a foramen in the TC (46). Kozerska and Skrzat (51) identified this foramen only in infant skulls and suggested that it transmits blood vessels to the vestibule or the superior vestibular nerve (SVN) via the AVA. They could not rule out that it may contain nerve fibers. Schart-Moren et al. (46) also found the TC channels using micro-CT. When the foramen was located above the crest, it ran mostly to the utricle, and, when it was situated beneath, it ran to the saccule. Here, the canal was traced either to the utricle or the saccule, or both, and continued along the nerve canals. In the saccule and utricle maculae, it sometimes ended at a distance from the foramina nervosa, suggesting that it contains a vessel. SR analyses, using a maximal intensity projection, occasionally showed an identifiable vessel. This is in concordance with Kozerska and Skrzat (51), who found that it may relay a branch or the AVA. It was earlier shown that selective obstruction of the AVA may be associated with sudden vertigo (52) followed by positional vertigo. Belal (53) found, after surgical damage of the AVA, a severe degeneration of the utricle and saccule maculae, as well as the cristae of the lateral and superior semi-circular canals (SSCCs), a finding that was verified experimentally by Silverstein and Makimoto (54). The clinical situation seems analogous to vestibular neuritis.

### Venous system

Venous drainage occurs via the vestibular and cochlear aqueducts. This organization is atypical, as it does not follow the arterial system. Venous branches have been described in the IAC, but their connection to the labyrinth is unclear (28,30). Cochlear blood is drained along the anterior and posterior spiral modiolar veins. From the vestibule, the anterior and posterior vestibular veins join the vein of the RW and drain into the ICV. The semicircular canal blood flows through the vein of the vestibular aqueduct and runs through a separate bone channel, known as the accessory canal of the VA or the para-vestibular canal (4). According to Perlman and Kimura (55), venous blockage leads to cochlear changes with reduced ganglion cells within two weeks in animal experiments. This suggests that collateral drainage between the cochlear and vestibular sides may be limited. These and the present results, which show that the ICV runs superficially in the floor of the ST near the RW, have clinical relevance. They infer that large CI electrodes can obstruct this important vein and, as a result, cause nerve degeneration. A 3D analysis also identified the vein of the RW which may be injured at cochleostomy drilling. The ICV had collateral veins draining from the middle ear. One such vein ran from a small ‘infundibulum’ in the floor of the RW niche (56). According to Watanabe et al. (57), these vessels may act as collateral veins following acute venous congestion of the inner ear.

Finally, although our inner ear research was originally considered academic, it has lately been translated into actions with more clinically relevant queries. Recent strategies, such as hearing preservation CI surgery and focusing on structural preservation and interventions in the IAC, provide additional awareness of the vascular supply in man. Micro-CT and SR imaging with 3D reconstructions substantiated the wide anatomic variations of the complex vascular anatomy, as earlier emphasized by investigators. Separation into arteries and veins was occasionally demanding, but was possible by using 3D rendering and cropping and by tracing back from identified vessels. The new techniques involving volume rendering may improve our understanding of the organization of the vascular system in the human labyrinth and equally illustrate its association with various inner ear disorders.

## Conclusion

Studies of the human inner ear are demanding because of the surrounding hard bone. However, in this study, for the first time, micro-CT and SR-PCI with 3D reconstructions were used to examine the major vascular tributaries in un-decalcified human temporal bone specimens. The extensive arterial supply along the plexus arteriosus was revealed. The results may help scientists to better understand vascular pathology and how it may affect inner ear function. The findings have important surgical implications.

## Disclosure statement

No potential conflict of interest was reported by the authors.

## Abbreviations

AVA: anterior vestibular artery
C: cochlea
CA: cochlear artery (or spiral modiolar artery)
CC: common crus
CI: cochlear implantation
CN: cochlear nerve
CNC: cochlear nerve canal
CVA: cochleo-vestibular artery (vestibular branch, cochlear branch)
CVN: cochlea-vestibular nerve
FC: facial canal
FN: facial nerve
FTC: foramen of the transverse crest
IAC: internal acoustic canal
ICV: inferior cochlear vein
IVN: inferior vestibular nerve
JB: jugular bulb
LA: labyrinthine artery
LP: labyrinthine portion of the facial canal
LSCC: lateral semi-circular canal
LW: lateral wall
OW: oval window
PSSC: posterior semi-circular canal
RW: round window
SiNC: singular nerve canal (canal for the posterior ampulla nerve)
SNC: saccular nerve canal
SR-PCI: synchrotron radiation phase contrast imaging
SS: sigmoid sinus
SSCC: superior semi-circular canal
ST: scala tympani
SV: scala vestibuli
SVN: superior vestibular nerve
SVNC: superior vestibular nerve canal
TC: transverse crest (or horizontal crest)
UNC: utricle nerve canal
V: vestibule
VA: vestibular aqueduct
VC: vertical crest (or “Bill’s bar”)
VCA: vestibular cochlear artery

## References

[1] Wilbrand HF, Rask-Andersen H, Gilstring D The vestibular aqueduct and the para-vestibular canal. An anatomic and roentgenologic investigation. Acta Radiol Diagn (Stockh). 1974;15:337–55. 4138016 10.1177/028418517401500401

[2] Rask-Andersen H, Stahle J, Wilbrand H Human cochlear aqueduct and its accessory canals. Ann Otol Rhinol Laryngol Suppl. 1977;86:1–16. 10.1177/00034894770860s501 410349

[3] Wadin K Imaging contributions to the temporal bone anatomy (high jugular fossae). Scand Audiol Suppl. 1988;30:145–8. 3227260

[4] Siebenmann F Die Korrosionsanatomie des knöchernen Labyrinthes des menschlichen Ohres. Wiesbaden, Germany: C. F. Bergmann; 1890.

[5] Siebenmann F Die Blutgefässa des Labyrinthes des menschlichen Ohres. Wiesbaden, Germany: C. F. Bergmann; 1894.

[6] Cotugno D Aquaeductibus auris humanae internae anatomica dissertatio. 1761.

[7] Atturo F, Barbara M, Rask-Andersen H On the anatomy of the 'hook' region of the human cochlea and how it relates to cochlear implantation. Audiol Neurootol. 2014;19:378–85. 25377867 10.1159/000365585

[8] Elfarnawany M, Rohani SA, Ghomashchi S, Allen DG, Zhu N, Agrawal SK, et al. Improved middle-ear soft-tissue visualization using synchrotron radiation phase-contrast imaging. Hear Res. 2017;354:1–8. 28822316 10.1016/j.heares.2017.08.001

[9] Agrawal S, Schart-Moren N, Liu W, Ladak HM, Rask-Andersen H, Li H The secondary spiral lamina and its relevance in cochlear implant surgery. Ups J Med Sci. 2018;123:9–18. 29537931 10.1080/03009734.2018.1443983 PMC5901472

[10] Kimura R, Perlman HB Arterial obstruction of the labyrinth. I. Cochlear changes. Ann Otol Rhinol Laryngol. 1958;67:5–24. 13521620 10.1177/000348945806700101

[11] Lawrence M Effects of interference with terminal blood supply on organ of Corti. Laryngoscope. 1966;76:1318–37. 5925174 10.1288/00005537-196608000-00003

[12] Zajtchuk JT, Falor WH Jr, Rhodes MF Hypercoagulability as a cause of sudden neurosensory hearing loss. Otolaryngol Head Neck Surg (1979). 1979;87:268–73. 503500

[13] Johnson A, Hawke M, Berger G Sudden deafness and vertigo due to inner ear hemorrhage–a temporal bone case report. J Otolaryngol. 1984;13:201–7. 6471153

[14] Axelsson A The vascular anatomy of the cochlea in the guinea pig and in man. Acta Otolaryngol. 1968:Suppl 243:3+. 5754509

[15] Axelsson A The cochlear blood vessels in guinea pigs of different ages. Acta Otolaryngol. 1971;72:172–81. 4939712 10.3109/00016487109122470

[16] Hawkins JE Jr, Johnsson LG, Preston RE Cochlear microvasculature in normal and damaged ears. Laryngoscope. 1972;82:1091–104. 5078635 10.1288/00005537-197207000-00001

[17] Schuknecht HF, Watanuki K, Takahashi T, Belal AA Jr, Kimura RS, Jones DD, et al. Atrophy of the stria vascularis, a common cause for hearing loss. Laryngoscope. 1974;84:1777–821. 4138750 10.1288/00005537-197410000-00012

[18] Thorne PR, Nuttall AL Laser Doppler measurements of cochlear blood flow during loud sound exposure in the guinea pig. Hear Res. 1987;27:1–10. 2953704 10.1016/0378-5955(87)90021-9

[19] Ohlsen KA, Didier A, Baldwin D, Miller JM, Nuttall AL, Hultcrantz E Cochlear blood flow in response to dilating agents. Hear Res. 1992;58:19–25. 1559902 10.1016/0378-5955(92)90004-7

[20] Nakashima T, Hattori T, Sone M, Asahi K, Matsuda N, Teranishi M, et al. Cochlear blood flow and speech perception ability in cochlear implant users. Otol Neurotol. 2012;33:165–8. 22215456 10.1097/MAO.0b013e318241c0db

[21] Dai M, Shi X Fibro-vascular coupling in the control of cochlear blood flow. PLoS One. 2011;6:e20652. 21673815 10.1371/journal.pone.0020652 PMC3106013

[22] Lee H, Whitman GT, Lim JG, Lee SD, Park YC Bilateral sudden deafness as a prodrome of anterior inferior cerebellar artery infarction. Arch Neurol. 2001;58:1287–9. 11493170 10.1001/archneur.58.8.1287

[23] Attanasio G, Cagnoni L, Masci E, Ciciarello F, Diaferia F, Bruno A, et al. Chronic cerebrospinal venous insufficiency as a cause of inner ear diseases. Acta Otolaryngol. 2017;137:460–3. 27846752 10.1080/00016489.2016.1252853

[24] Fukuda R, Miyamoto N, Hayashida A, Ueno Y, Yamashiro K, Tanaka R, et al. Acute hearing loss caused by decreasing anterior inferior cerebellar arterial perfusion in a patient with vertebral artery stenosis. J Stroke Cerebrovasc Dis. 2017;26:e119–21. 28372948 10.1016/j.jstrokecerebrovasdis.2017.03.030

[25] Lu YY, Jin Z, Tong BS, Yang JM, Liu YH, Duan M A clinical study of microcirculatory disturbance in Chinese patients with sudden deafness. Acta Otolaryngol. 2008;128:1168–72. 19241603 10.1080/00016480801901626

[26] Ohlemiller KK, Rice ME, Gagnon PM Strial microvascular pathology and age-associated endocochlear potential decline in NOD congenic mice. Hear Res. 2008;244:85–97. 18727954 10.1016/j.heares.2008.08.001 PMC2630541

[27] Johnsson LG, Hawkins JE Jr. Vascular changes in the human inner ear associated with aging. Ann Otol Rhinol Laryngol. 1972;81:364–76. 4113137 10.1177/000348947208100307

[28] Shambaugh G Blood vessels in the labyrinth of the ear. Vol.10 decennial publication. Chicago: University of the Chicago Press; 1903.

[29] Asai K Die Blutgerisse des heutigen Labyrinthes der Ratte. Anatomische Hefte. 1908;36:711.

[30] Asai K Die Blutgefisse im heutigen Labyrinthe des Hundes. Anatomische Hefte. 1908;36:369.

[31] Charachon R Anatomie de Parthe auditive interne chez Phomme. Lyon: Imprimerie Bosc Frhres; 1961.

[32] Agazzi C Appunti di idrodinamica vascolore del legemento spirale, Arch ital di otol. 1949;60:40–7.

[33] Smith CA Capillary areas of the membranous labyrinth. Ann Otol Rhinol Laryngol. 1954;63:435–47. 13189309 10.1177/000348945406300213

[34] Levin NA Die Vaskularisation des Ohrlabyrinthes beim Menschen. Anat Anz. 1964;114:337. 14211472

[35] Scuderi R, Bo MD La vascolarizzazione del labirinto umano. Arch di Otol Rinol e Laringol. 1952;63:Suppl11. 14944402

[36] Mazzoni A Internal auditory canal arterial relations at the porus acusticus. Ann Otol Rhinol Laryngol. 1969;78:797–814. 5799404 10.1177/000348946907800413

[37] Mazzoni A Internal auditory artery supply to the petrous bone. Ann Otol Rhinol Laryngol. 1972;81:13–21. 5009813 10.1177/000348947208100103

[38] Fisch U The surgical anatomy of the so called internal auditory artery In: Hamberger CA, Wersfill J, editors. Disorders of the skull base region. New York: J. Wiley and Sons; 1969 p. 121–30.

[39] Tange RA, Hodde KC Microvasculature of the stria vascularis in the round window area in the rat. A scanning electron microscopy study. ORL J Otorhinolaryngol Relat Spec. 1985;47:225–8. 4047623 10.1159/000275776

[40] Schwalbe G Ein Beitrag zur Kenntnis der Circulationsverhältnisse in der Gehörschnecke In: Ludwig C., editor Beiträge zur Physiologie. Festschrift. Leipzig: F. C. W. Vogel; 1887 p. 200–20.

[41] Eichler O Anatomische Untersuchungen iiber die Wege des Blutstromes im menschlichen Ohrlabyrinth. Abhandl. der mathem.-phys. Classe der konigtich suchsischen Gesellschaft der Wissenschaften. 1892;18:310.

[42] Nabeya D A study in the comparative anatomy of the blood-vascular system of the internal ear in Mammalia and Homo. Acta Scholae Medicinalis, Kyoto University. 1923;6:1–132.

[43] Fedorov A, Beichel R, Kalpathy-Cramer J, Finet J, Fillion-Robin JC, Pujol S, et al. 3D slicer as an image computing platform for the Quantitative Imaging Network. Magn Reson Imaging. 2012;30:1323–41. 22770690 10.1016/j.mri.2012.05.001 PMC3466397

[44] Koch RW, Elfarnawany M, Zhu N, Ladak HM, Agrawal SK Evaluation of cochlear duct length computations using synchrotron radiation phase-contrast imaging. Otol Neurotol. 2017;38:e92–9. 28595256 10.1097/MAO.0000000000001410

[45] Wysokinski TW, Chapman D, Adams G, Renier M, Suortti P, Thomlinson W Beamlines of the biomedical imaging and therapy facility at the Canadian light source - part 3. Nucl Instrum Meth A. 2015;775:1–4.

[46] Schart-Moren N, Larsson S, Rask-Andersen H, Li H Anatomical characteristics of facial nerve and cochlea interaction. Audiol Neurootol. 2017;22:41–9. 28628917 10.1159/000475876

[47] Rask-Andersen H, Li H, Lowenheim H, Muller M, Pfaller K, Schrott-Fischer A, et al. Supernumerary human hair cells-signs of regeneration or impaired development? A field emission scanning electron microscopy study. Ups J Med Sci. 2017;122:11–19. 28145795 10.1080/03009734.2016.1271843 PMC5361427

[48] Liu W, Li H, Edin F, Brannstrom J, Glueckert R, Schrott-Fischer A, et al. Molecular composition and distribution of gap junctions in the sensory epithelium of the human cochlea-a super-resolution structured illumination microscopy (SR-SIM) study. Ups J Med Sci. 2017;122:160–70. 28513246 10.1080/03009734.2017.1322645 PMC5649321

[49] Malan E Dei vasi glomerulari del labirinto osseo. Boll Soc ital Biol sper. 1931;6:832–4.

[50] Balogh K, Koburg E Der Plexus cochlearis. Arch Ohr Nus Kehlkopfheilk. 1965;185:638. 10.1007/BF02148945 5881096

[51] Kozerska M, Skrzat J Anatomy of the fundus of the internal acoustic meatus - micro-computed tomography study. Folia Morphol (Warsz). 2015;74:352–8. 26339817 10.5603/FM.2015.0053

[52] Lindsay J, Hemenway W LVII postural vertigo due to unilateral sudden partial loss of vestibular function. Ann Otol Rhinol Laryngol. 1956;65:692. 13363229 10.1177/000348945606500311

[53] Belal A Jr. The effects of vascular occlusion on the human inner ear. J Laryngol Otol. 1979;93:955–68. 512467 10.1017/s0022215100087958

[54] Silverstein H, Makimoto K Superior vestibular and “singular nerve” section–animal and clinical studies. Laryngoscope. 1973;83:1414–32. 4758756 10.1288/00005537-197309000-00004

[55] Perlman HB, Kimura R Experimental obstruction of venous drainage and arterial supply of the inner ear. Ann Otol Rhinol Laryngol. 1957;66:537–46. 13459249

[56] Atturo F, Schart-Moren N, Larsson S, Rask-Andersen H, Li H The human cochlear aqueduct and accessory canals: a micro-CT analysis using a 3D reconstruction paradigm. Otol Neurotol. 2018;39:e429–35. 29794687 10.1097/MAO.0000000000001831

[57] Watanabe Y, Nakashima T, Yanagita N Venous communications of the cochlea after acute occlusion of the vein of the cochlear aqueduct. Arch Otorhinolaryngol. 1988;245:340–3. 3248070 10.1007/BF00457990

